# Comparing 12-core and 20-core biopsy for prostate cancer diagnosis with transperineal MR/US fusion biopsy: assessing the effective number of systemic cores using propensity score matching

**DOI:** 10.1007/s11255-023-03674-2

**Published:** 2023-06-20

**Authors:** Hyeok Jae Kwon, Seung Ah Rhew, Chang Eil Yoon, Dongho Shin, Seokhwan Bang, Yong Hyun Park, Hyuk Jin Cho, U-Syn Ha, Sung-Hoo Hong, Ji Youl Lee, Sae Woong Kim, Hyong Woo Moon

**Affiliations:** Department of Urology, College of Medicine, Seoul St. Mary’s Hospital, The Catholic University of Korea, 222, Banpo-daero, Seocho-gu, Seoul, 06591 Republic of Korea

**Keywords:** Prostate cancer, Propensity score, Image-guided biopsy, Magnetic resonance imaging

## Abstract

**Purpose:**

For transperineal (TP) prostate biopsy, target biopsy for visible lesions on MRI is important, but there is no consensus of the number of systemic biopsy cores. Our study aimed to confirm the diagnostic efficiency of 20-core systemic biopsy by comparison with 12-core using propensity score matching (PSM).

**Methods:**

The 494 patients conducted the naive TP biopsy were retrospectively analyzed. There were 293 patients with 12-core biopsy and 201 patients with 20-core biopsy. PSM was performed for minimizing confounding variables, and the established effects’ value was analyzed for ‘index-positive or negative’ clinically significant prostate cancer (csPCa) (Index means PIRADS Score ≥ 3 on multiparametric prostate MRI).

**Results:**

At 12-core biopsy, there were 126 cases of prostate cancer (43.0%), and 97 cases of csPCa (33.1%). At 20-core biopsy, there were 91 cases (45.3%) and 63 cases (31.3%). After propensity score matching, for index-negative csPCa, the estimated odds ratio was 4.03 (95% CI 1.35–12.09, *p* value 0.0128), and for index-positive csPCa, the estimated odds ratio was 0.98 (95% CI 0.63–1.52, *p* value 0.9308).

**Conclusions:**

The 20-core biopsy did not show a higher detection rate for csPCa in comparison with the 12-core biopsy. However, when MRI did not show a suspicious lesion, 20-core biopsy showed higher odd ratio in comparison with 12-core biopsy. Therefore, if there is a suspicious lesion in MRI, 20-core biopsy is excessive and 12-core biopsy is sufficient. Whereas if there is no suspicious lesion in MRI, it is better to proceed with 20-core biopsy.

## Introduction

Worldwide, prostate cancer is the third most common cancer, with an estimated 1,414,259 new cases and 375,304 deaths reported in 2020 [1]. In South Korea, prostate cancer is rapidly increasing and ranks as the fourth most common cancer among males, with estimated 16,749 new cases and 2140 deaths reported in 2021 [2]. For the histological diagnosis of prostate cancer, transrectal ultrasonography (TRUS) biopsy using a 12-core biopsy template was considered the standard procedure in the past [3]. However TRUS biopsy showed a high false-negative rate (one study showed that it was up to 30% [4]) and several complications were observed. Some of the complications were severe enough to require patient hospitalization [5]. In particular, infection through the rectum by TRUS biopsy can induce sepsis, so European Association of Urology (EAU) and American Urology Association (AUA) recommend transperineal biopsy if possible [6].

Multiparametric magnetic resonance imaging (MRI) can provide novel functional parameters for non-invasive risk assessment before biopsy [7], so it becomes a very important tool for prostate cancer’s initial diagnosis [8, 9]. Therefore, transperineal biopsy using multiparametric MRI is recommended for prostate cancer biopsy recently, because of the low false-negative rate and decreased unnecessary biopsy [10]. And, it also showed improved sensitivity for detecting intermediate to high-risk prostate cancer and decreased over-detecting of low-risk prostate cancer [9].

The use of MRI along with target biopsy is shown to be more effective in detecting clinically significant prostate cancer (csPCa) than using the classical pathway of systemic biopsy alone [11]. However, there is no established guideline regarding the number of systemic biopsy cores that should be performed. Some studies assert that the combination of systemic and target biopsy detects additional csPCa in 4.3–5.2% of patients but results in a higher rate of overdiagnosis of insignificant prostate cancer [12]. On the other hand, other studies assert that csPCa was found in 10 to 40% when prostate-specific antigen (PSA) was high, but there was no lesion on MRI [Prostate Imaging-Reporting and Data System (PIRAD) Score ≤ 2] at those cases [13, 14]. In both opposing perspectives, a common point is the importance of appropriate number of systemic biopsy cores. For TRUS biopsy, it has already been proven by several studies that 12-core systemic biopsy enables accurate risk stratification with reducing repeat biopsies and not increasing insignificant cancer detection compared to previous sextant biopsy method [10]. Furthermore, conducting more than 12 systemic cores does not aid in diagnosing csPCa than 12 cores, and is not recommended for raising the insignificant cancer detection rate [15].

However, for transperineal biopsy, several studies have been conducted only focused on the target biopsies, but no establishment has been made on the appropriate number of systemic biopsy cores. Therefore, our study aim to investigate the optimal number of systematic cores during transperineal biopsy and identify the situations where their application would be most beneficial.

## Method

### Patients and data

Before the analysis, our study established inclusion criteria to minimize bias. The inclusion criteria consisted of three factors: no prior history of prostate biopsy, no prior use of a 5-alpha reductase inhibitor, and PSA levels ≥ 3 ng/ml. This study retrospectively analyzed a total of 494 patients, who underwent transperineal prostate biopsy at Seoul St. Mary's Hospital, Korea, between June 2020 and June 2021. Among these 494 patients who underwent transperineal biopsy, 293 patients underwent a 12-core systemic biopsy, while 201 patients underwent a 20-core systemic biopsy.

PSA values within 3 months prior to the biopsy were selected, and in cases of multiple values, the PSA value closest to the biopsy day was chosen. Prostate MRI was performed for all patients within 3 months before the biopsy. The MRI scans were interpreted by a radiologist at Seoul St. Mary's Hospital. The prostate volume was measured using MRI and calculated using the formula “height × width × length × 0.52”[16, 17]. When a lesion on MRI had a PIRADS score of 3 or higher, we categorized it as “Index positive.” Conversely, if there was no lesion or a lesion with a PIRADS score of 1–2, we classified it as “Index negative.”

The biopsy specimens were examined and evaluated by a pathologist specialized in urologic pathology at Seoul St. Mary’s Hospital. In this study, prostate cancer cases with a Gleason score 7 (3 + 4) or higher were categorized as csPCa.

### Transperineal biopsy technique

All patients were advised to discontinue anticoagulants for a minimum of 7 days before and after the prostate biopsy. The biopsy procedure took place in the operating room, and the patients underwent either laryngeal mask airway (LMA) anesthesia or monitored anesthesia care (MAC). Following anesthesia, prophylactic intravenous antibiotics were administered. Cefotetan, a second-generation cephalosporin, was selected as the primary choice for prophylactic antibiotics. However, in cases where patients exhibited skin allergic reactions, levofloxacin was used as an alternative antibiotic. After anesthesia, patients were positioned in the lithotomy position, and the perineum was prepared using betadine dressing.

Next, we used a transrectal ultrasound (BK-5000) for guidance during the procedure. The “BioJet™ fusion software program” was employed for MRI-ultrasound matching. When a lesion with a PIRADS score 3 or higher was detected, it was designated as a target, and a target biopsy was conducted as the first step. If there was only one target lesion, a 3-core biopsy was performed for that specific target. However, if there were multiple targets, a 2-core biopsy was performed for each target. For instance, if a patient's MRI revealed two PIRADS 4 lesions, the target biopsy would involve a total of four cores.

**Fig. 1:**
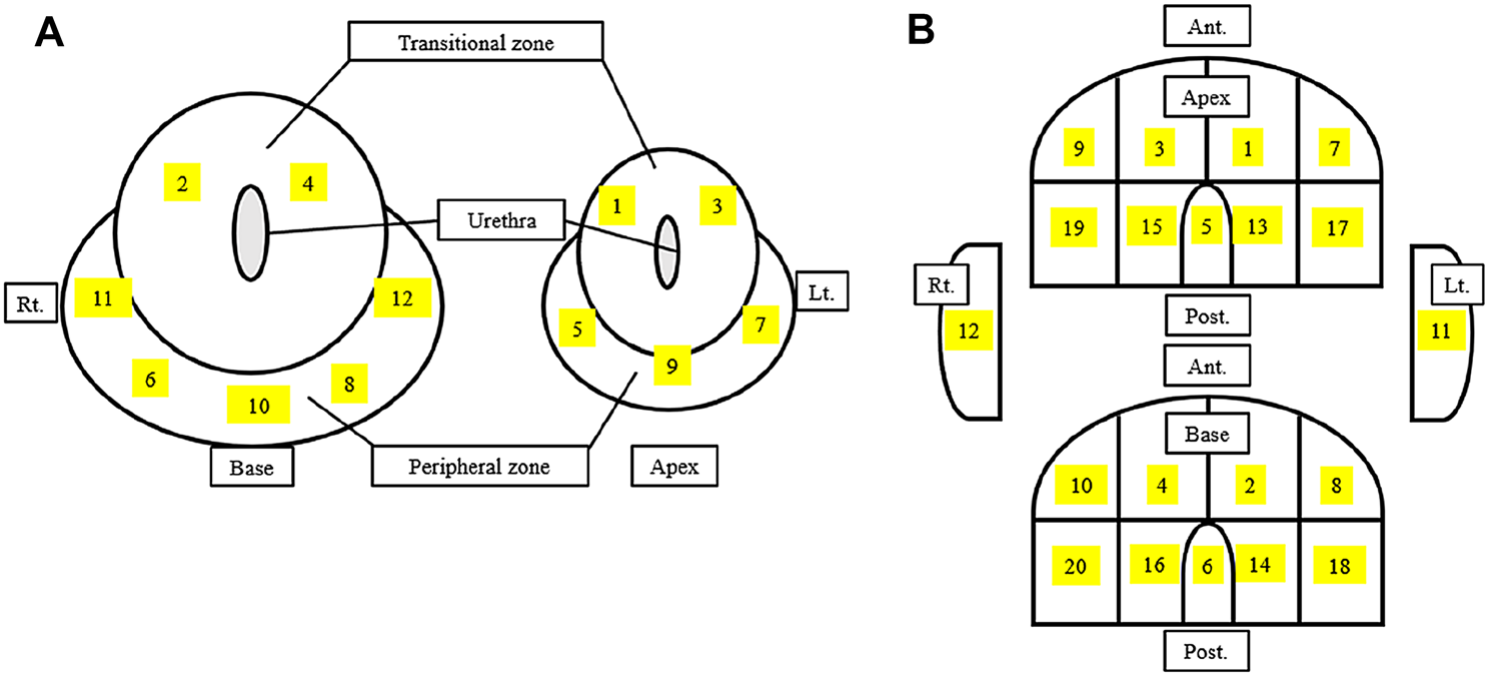
Biopsy template: 12-core and 20-core. **A** 12-cores transperineal prostate biopsy template of Dr. Shoji research: Each transitional zone apex and base was collected as (1)–(4), and each peripheral zone apex and base was collected as (5)–(8). Dorsal peripheral zone apex & base were collected as (9) and (10), and both peripheral zone lateral sides were collected as (11) and (12). **B** 20-cores transperineal prostate biopsy template of the modified Barzell zone: Zones (1)–(4) and (7)–(10) were collected as the anterior prostate, but on opposite, zones (13)–(16) and (17)–(20) were collected as the posterior prostate. Zones (5) and (6) were collected as the midline of posterior prostate

Following the target biopsy, a systemic biopsy was conducted using either a 12-core template or a 20-core template. There were no specific criteria for determining which patients would undergo a 12-core biopsy or a 20-core biopsy. For the 12-core biopsy cases, the transperineal prostate biopsy template developed by Dr. Shoji's research was used [18]. On the other hand, for the 20-core biopsy cases, the modified Barzell zone template was used [19] (Fig. 1).

The biopsy procedures were performed by two experienced urologists, each having conducted over 100 cases of transperineal biopsy. After the procedure, the biopsy site was sterilized with betadine. Patients were then monitored in the recovery room for 30 min after regaining consciousness from anesthesia. Upon recovery, patients were discharged without a Foley catheter if they did not experience any urination-related issues. Between 7 and 10 days after the biopsy, patients attended a follow-up appointment at the outpatient clinic, during which they were asked about any complications that occurred post-biopsy. Significant complications were defined as gross hematuria requiring hospitalization in the emergency room or another medical facility, voiding difficulties necessitating foley catheter insertion, and a fever of 37.8 degrees Celsius or higher.

### Statistical analysis

The Chi-square test and *T* test were employed to analyze the continuous variables between the two groups. The statistical analysis was performed using the R version 4.1.0 as the software. A *p* value less than 0.05 was considered statistically significant. To account for confounding variables, propensity score matching (PSM) was utilized to estimate the average marginal effect of the 20-core biopsy on prostate cancer in the population that underwent the procedure.

Full matching on the propensity score was attempted to achieve satisfactory balance between the groups. After the matching process, it was observed that all standardized mean differences for the covariates were below 0.1, indicating a satisfactory balance between the groups. The propensity score was estimated using logistic regression of the 20-core biopsy group on the covariates. In the full matching approach, both the 12-core and 20-core biopsy groups were used, ensuring that no units were discarded during the matching process.

## Result

A comparison was conducted between 293 patients who underwent 12-core biopsy and 201 patients who underwent 20-core biopsy. The demographic features of each group are presented in Table 1. The average age of the 12-core group was 68.2 years, which was significantly higher than the average age of the 20-core group, which was 66.7 years (*p* value 0.036). In terms of prostate volume, the average volume of the 12-core group was 44.8 cc, which was significantly smaller than the 50.4 cc of the 20-core group (*p* value 0.007). Regarding the presence of index lesions, 242 out of 293 (82.6%) patients in the 12-core group had index lesions, whereas 105 out of 201 (52.2%) patients in the 20-core group had index lesions (*p* value < 0.001). Among the patients who underwent the 12-cores biopsy, 2 patients (0.7%) experienced gross hematuria, and 1 patient (0.4%) experienced acute urinary retention (AUR). In contrast, among the patients who underwent the 20-cores biopsy, 3 patients (1.4%) experienced gross hematuria, and 3 patients (1.4%) experienced AUR. The *p* value for the comparison of complication rates between the two groups was 0.25, indicating no statistically significant difference.

**Table 1:** Demographic data and post-biopsy complication for each group

Group	12-core	20-core	*p* value
	(*N* = 293)	(*N* = 201)	
Age	68.2 ± 8.0	66.7 ± 7.9	0.036
All prostate cancer	126 (43.0%)	91 (45.3%)	0.684
csPCa	97 (33.1%)	63 (31.3%)	0.754
PSA (ng/ml)	11.5 ± 45.9	17.4 ± 52.5	0.196
Prostate volume (cc)	44.8 ± 22.1	50.4 ± 23.9	0.007
Index lesion (+)	242 (82.6%)	105 (52.2%)	< 0.001
Index lesion (−) csPCa	12 (4.1%)	20 (10.0%)	0.016
Target PIRADS score			< 0.001
2	9 (3.1%)	0 (0.0%)	
3	46 (15.7%)	29 (14.4%)	
4	126 (43.0%)	32 (15.9%)	
5	61 (20.8%)	44 (21.9%)	
Gleason score			0.271
6 (3 + 3)	40 (13.7%)	28 (13.9%)	
7 (3 + 4)	42 (14.3%)	33 (16.4%)	
7 (4 + 3)	38 (13.0%)	20 (10.0%)	
8 (4 + 4)	14 (4.8%)	4 (2.0%)	
9–10	3 (1.0%)	6 (3.0%)	
Complications	3 (1.1%)	6 (2.8%)	0.250
Hematuria	2 (0.7%)	3 (1.4%)	
Acute urinary retention	1 (0.4%)	3 (1.4%)	

Due to the retrospective nature of the comparison, there were factors that exhibited significant differences between the two groups, which could potentially introduce selection bias. Therefore, they were set as covariates and PSM was conducted. As a result of biopsy, the rates of prostate cancer in the two groups were 126 out of 293 (43.0%) and 91 out of 201 (45.3%), respectively. The *p* value for this comparison was 0.684, indicating no significant difference between the groups in terms of prostate cancer detection. The rates of csPCa in the 12-core and 20-core biopsy groups were 97 out of 293 (33.1%) and 63 out of 201 (31.3%), respectively, with a *p* value of 0.754, signifying no statistically significant difference in the rates of csPCa between the two groups.

**Fig. 2:**
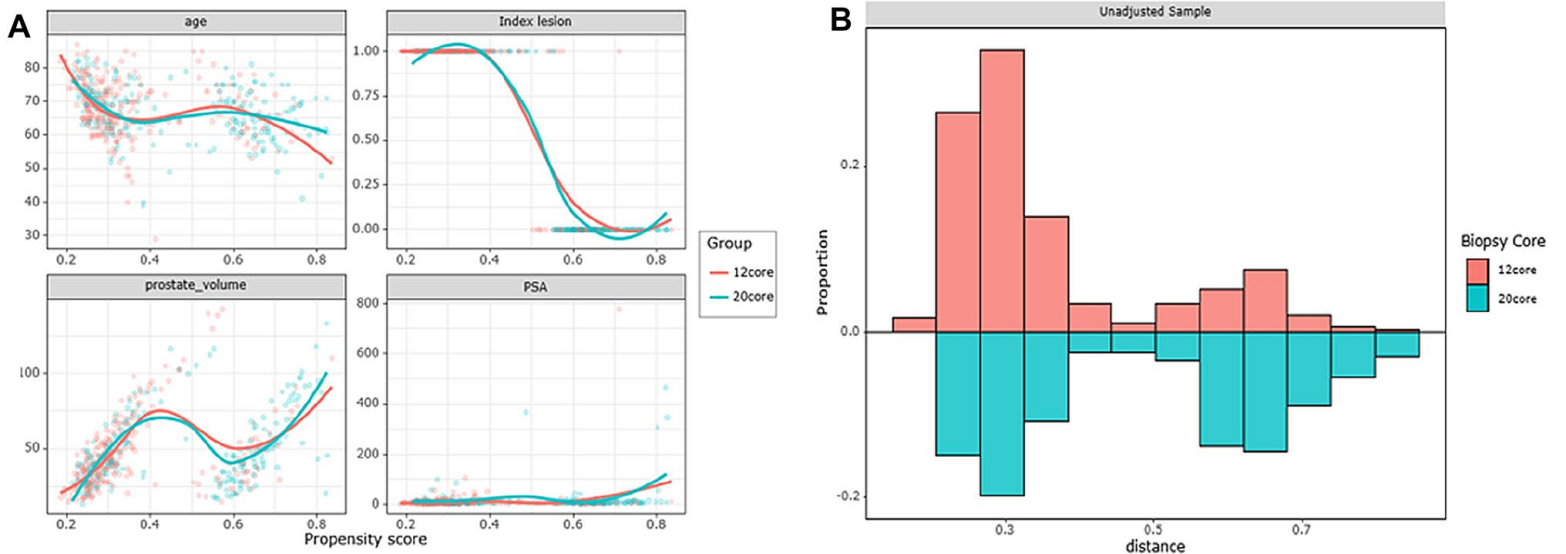
Covariates before and after PSM, and the distance distribution. **A** Covariates before and after PSM: when compared with the spread data before PSM, the difference between covariates decreased after PSM. **B** Statistical distance: calculated by covariates of each group`s patients before PSM

**Fig. 3:**
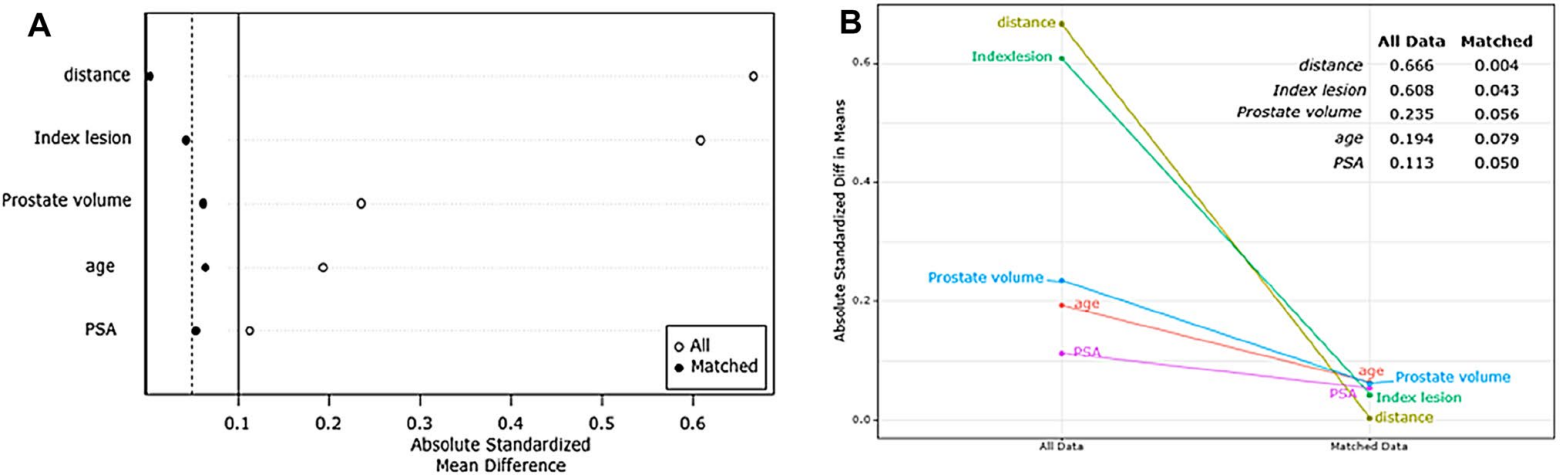
Changes of absolute standardized differences. **A** Love plot: showing the statistical deference (SD) of each covariate after PSM, **B** Graph for change of SD: All covariates have SD values less than 0.1 (if the SD value after PSM is less than 0.1, it can be said that the difference in covariates has been reduced well and as shown in the figures)

Figure 2A presents a graph illustrating the distribution of covariates before and after PSM. The comparison reveals a reduction in the differences between covariates after PSM, as observed by the narrower spread of data compared to the pre-PSM stage. In Fig. 2B, the statistical distance between patient covariates is displayed, calculated before PSM. Figure 3A presents a love plot showing the statistical deference (SD) of each covariate after PSM. The graphical representation of these differences can be seen in Fig. 3B. It is noteworthy that all covariates exhibit SD values below 0.1, indicating a significant reduction in covariate differences. To summarize the findings, Table 2 provides a comprehensive overview by combining the information presented in both figures.

**Table 2:** Propensity score matching to estimate the average marginal effect of the 20-core biopsy on prostate cancer

Variables	Before propensity score matching				After propensity score matching			
	12-core	20-core	*p* value	Standardized difference	12-core	20-core	*p* value	Standardized difference
Covariates	*N* = 293	*N* = 201			*N* = 293	*N* = 201		
Age (years)	68.2 ± 8.0	66.7 ± 7.9	0.036	− 0.194	66.0 ± 7.8	66.7 ± 7.9	0.388	0.079
PSA (ng/ml)	11.5 ± 45.9	17.4 ± 52.5	0.196	0.113	20.0 ± 93.7	17.4 ± 52.5	0.718	− 0.050
Prostate volume (cc)	44.8 ± 22.1	50.4 ± 23.9	0.007	0.235	51.7 ± 27.2	50.4 ± 23.9	0.573	− 0.056
Index lesion	82.6%	52.2%	< 0.001	− 0.608	54.4%	52.2%	0.738	− 0.043

^a^Values are weighted mean ± weighted SD or weighted percentages

After minimizing the covariate disparities through PSM, the rates of csPCa diagnosis were compared between the two groups, as presented in Table 3. When an index lesion was observed on the MRI, the odds ratio of 20-cores biopsy to 12-cores was 0.98, with a *p* value of 0.9, indicating no significant difference between the two groups. However, in cases where no index lesion was detected, the odds ratio of 20-cores biopsy to 12-cores was 4.03, with a *p* value of 0.013, demonstrating a statistically significant difference.

**Table 3:** Odds ratio according to the presence of an index lesion in detecting csPCa using 20-core biopsy

	Clinically significant prostate cancer			
	Odd ratio	95% CI	*p* value	
Index (+)	0.98	0.63–1.52	0.9308	
Index (−)	4.03	1.35–12.09	0.0128	

## Discussion

As the incidence and prevalence of prostate cancer increase, the importance of diagnostic biopsies has also grown. While transrectal approach was previously common, transperineal approach is now recommended for its higher accuracy and safety. Consequently, numerous studies have been conducted and are currently underway. However, most of the researches on transperineal biopsies have focused on target biopsy, while there has been limited investigation into systemic biopsy cores outside the target lesion. To the best of the author’s knowledge, this is the first study to compare the effects of systemic core number on transperineal biopsy.

In this study, the patients undergoing transperineal biopsy were divided into two groups based on the presence or absence of lesions with a PIRADS score of 3 or higher on MRI. Among the patients with lesions observed on MRI, there was no statistically significant difference in the diagnosis rate of csPCa between the 12-core and 20-core systemic biopsy groups. However, when no lesions were observed on MRI or when a lesion with a PIRADS score of less than 3 was present, the 20-core systemic biopsy demonstrated a significantly higher odds ratio for the diagnosis of csPCa compared to the 12-core systemic biopsy.

It is important to note that in the case of transrectal biopsy, the complication rate tends to increase with the number of biopsy cores due to the increased risk of infection and rectal bleeding. Similarly, in other study of transperineal biopsy, the complication rate generally increases as the number of biopsy cores increases [20]. However, in our study, there was no significant difference in the incidence of complications between 12-core biopsy and 20-core biopsy. And our study also showed relatively low complications at 1.1% for 12-core and 2.8% for 20-core than other studies.

However, our study had several limitations. First, due to its retrospective nature, the assignment of patients to the 12-core and 20-core biopsy groups was not randomized, resulting in significant differences in age, prostate volume, and PIRADS score between the two groups. To mitigate this potential source of statistical error, we employed the PSM method. Additionally, the sample size of 494 patients may be considered relatively small, which could limit the generalizability of our findings. Second, we did not consider the volume of the prostate in our analysis. In a study conducted by Dr. Shoji, high-intensity focused ultrasound (HIFU) treatment was performed following a 12-core biopsy, and it was found that 8.9% of the follow-up biopsy results failed to detect csPCa. The failure in detection was attributed to selection bias based on prostate size [18]. The Ginsburg study also suggests the number of systemic biopsy cores according to size for standardization of transperineal biopsy [21]. In our study, the prostate volume was 44.8 ± 22.1 in the 12-core biopsy group and 50.4 ± 23.9 in the 20-core group (*p* value 0.007), indicating that the 20-core group had a significantly larger prostate volume, but the analysis according to groups based on prostate volume was not performed. Third, the criteria for complications were based on the requirement of inpatient treatment or visits to other hospitals, and the assessment of the severity of complications is subjective, as it relies on the patients' subjective judgment. Fourth, in patients suspected of prostate cancer, MRI was performed before biopsy as the first step of cancer evaluation. However the cost and accessibility of MRI vary according to each country, and the protocol of this study that MRI must be taken before biopsy can be seen as a limitation.

## Conclusion

While many studies have highlighted the importance of transperineal biopsy for targeted biopsies, the significance of systemic biopsy cores in comparison to previous transrectal biopsy standards has received relatively less attention. In this study, our focus was specifically on analyzing the results of systemic biopsy cores while excluding pathologic result of target biopsies. The findings suggest that when an MRI reveals a lesion with a PIRADS score of 3 or higher, a 12-core systemic biopsy is sufficient, as there is no significant difference compared to a 20-core biopsy in diagnosing csPCa. However, if there is a lesion with a PIRADS score below 3 or no lesion observed, a 20-core biopsy is preferable over a 12-core biopsy for diagnosing csPCa. It is important to note that there were no significant differences in complication rates associated with an increase in the number of systemic biopsy cores.

## Data Availability

Data can be made available upon reasonable request.
